# The negative effect of initial high-dose methylprednisolone and tapering regimen for acute respiratory distress syndrome: a retrospective propensity matched cohort study

**DOI:** 10.1186/s13054-017-1723-0

**Published:** 2017-06-08

**Authors:** Makoto Takaki, Kazuya Ichikado, Kodai Kawamura, Yasuhiro Gushima, Moritaka Suga

**Affiliations:** Division of Respiratory Medicine, Saiseikai Kumamoto Hospital, 5-3-1 Chikami Minami-ku, Kumamoto city, Kumamoto 861-4193 Japan

**Keywords:** Acute respiratory distress syndrome, High-dose corticosteroid therapy, Mortality

## Abstract

**Background:**

The efficacy of corticosteroid use in acute respiratory distress syndrome (ARDS) remains controversial. Generally, short-term high-dose corticosteroid therapy is considered to be ineffective in ARDS. On the other hand, low-dose, long-term use of corticosteroids has been reported to be effective since they provide continued inhibition of the systemic inflammatory response syndrome (SIRS) that accompanies ARDS. Thus far, no reports have been published on the efficacy of initiating treatment with a high-dose corticosteroid regimen with tapering.

**Methods:**

We conducted a retrospective observational study involving 186 patients treated at a teaching hospital (68% had sepsis, pneumonia, or aspiration pneumonia). ARDS was diagnosed according to the Berlin definition. Patients were divided into a high-dose (n = 21) or low-dose corticosteroid group (n = 165) to compare the effectiveness of a down-titration regimen. The primary medical team chose which treatment a patient would receive. We were careful to conduct a differential diagnosis of interstitial pneumonia (e.g., acute eosinophilic pneumonia) since corticosteroid treatment has been proven effective in that patient population. The primary outcome was the 60-day mortality rate. The secondary outcome was the number of ventilator-free days (VFD).

**Results:**

Those started on a high-dose regimen had a significantly higher 60-day mortality rate (*P* = 0.031) with significantly fewer VFD (*P* = 0.021). Propensity scores were used to adjust patient backgrounds in a variable analysis that also showed the high-dose regimen was a factor in decreasing VFD (OR, 95.63; 95% CI, 1.74–5271.07; *P* = 0.026) and worsening the 60-day mortality rate (OR, 2.54; 95% CI, 0.92–7.02; *P* = 0.072).

**Conclusions:**

A tapering regimen after high-dose corticosteroids is likely to increase ventilator dependency and might aggravate the prognosis of patients with ARDS diagnosed according to the Berlin definition.

## Background

Acute respiratory distress syndrome (ARDS) is lung injury resulting from excessive inflammation induced by various causes [1]. Drugs with anti-inflammatory effects have been expected to be effective in treating ARDS [2]. Most research thus far has looked primarily at the therapeutic efficacy of corticosteroids, which have a potent anti-inflammatory effect [3–7], but the efficacy of corticosteroids in ARDS is yet to be established. Moreover, accumulated evidence has ruled out the effectiveness of short-term, high-dose corticosteroid therapy [8–11]. However, low-dose, long-term corticosteroid use has been reported as effective in providing continuous control of the systemic inflammatory response syndrome (SIRS) that accompanies ARDS [4]. On the other hand, administration of 1 g/day of methylprednisolone for 3 days followed by tapering methylprednisolone-pulse therapy has been reported to be effective in serious disease states due to immune disorders such as IgA nephropathy [12]. Initial high-dose corticosteroid treatment is given a weak recommendation for use in acute exacerbation of an ARDS-like disease state under the International Guidelines for Idiopathic Pulmonary Fibrosis [13]. In East Asia, it is not uncommon for high-dose corticosteroid treatment to be used in serious respiratory failure. In disease states that fulfill the diagnostic criteria for ARDS, there are cases of interstitial pneumonitis in which corticosteroids can be effective, such as acute eosinophilic pneumonia and acute organizing pneumonia [14, 15], and since these disease states cannot be ruled out in clinical practice, high-dose corticosteroid therapy may be instituted. In ARDS, high-dose corticosteroids are initially administered followed by a tapering regimen, but the efficacy of this treatment has not been reported. We divided our patients into two groups that received either high initial doses or low doses to examine the effectiveness of a tapering regimen and through propensity score analyses we conducted a retrospective comparative investigation.

## Methods

### Patients

We collected data on patients diagnosed with ARDS, who were treated at our institution between 1 October 2004 and 30 June 2015, in a retrospective observational study. ARDS was diagnosed according to the Berlin definition [16]. Patients with a medical history or imaging findings indicating chronic fibrotic interstitial pneumonia were excluded. In addition, patients definitively diagnosed with conditions ordinarily treated with corticosteroids, such as diffuse pulmonary alveolar hemorrhage due to vasculitis syndrome, acute eosinophilic pneumonia, and acute hypersensitivity pneumonitis, were also excluded. These diagnoses were conducted comprehensively based on medical history, blood tests, and findings on imaging and bronchoalveolar lavage (BAL). Diagnoses of steroid-responsive interstitial pneumonia, such as acute hypersensitivity pneumonitis or acute organizing pneumonia, were based on antigen exposure history, high-resolution computed tomography (HRCT) findings, cellular pattern and CD4/CD8 ratio of BAL. In acute hypersensitivity pneumonitis, in addition to environmental factors, characteristic HRCT findings of patchy ground-glass opacity and small centrilobular nodular opacity and BAL lymphocytosis with low CD4/CD8 ratio were noted. Informed consent was obtained in writing from patients and their families.

### Data collection and definitions

At the time of diagnosis of ARDS, age, sex, McCabe classification [17], arterial oxygen tension/fraction of inspired oxygen (PaO_2_/FiO_2_) ratio, severity according to the Berlin definition [16], and the cause of the ARDS were recorded. Causes of ARDS were classified as direct injury, indirect injury, infectious disease, or non-infectious disease. Blood test data, including white blood cell counts, platelet counts, C-reactive protein (CRP), serum albumin, and lactate dehydrogenase (LDH) were recorded.

The Acute Physiology and Chronic Health Evaluation II score (APACHE II score) [18] and the Sequential organ failure assessment score (SOFA score) [19] were recorded as indicators of disease severity at diagnosis. Diagnosis of ventilator-associated pneumonia (VAP) was determined based on the following criteria: onset 48 hours or more after the start of respirator use, radiography and computed tomography (CT) of the chest showing evidence of new opacities and pulmonary edema could be ruled out as a cause of the opacity, suctioned sputum culture tests revealing >10^7^ colony-forming units (cfu)/mL of bacteria, or gram staining revealing local phagocytosis.

HRCT images were obtained in all cases at the time of diagnosis. Semi-quantitative assessment of fibroproliferative changes using HRCT scans was used to determine scores (HRCT score, hereinafter). Previously, we reported that this score could be used as an independent factor to predict the prognosis of ARDS [20]. In that report, high HRCT scores reflected the fact that fibroproliferative changes are widespread, susceptibility to mechanical ventilation-related lung injury is enhanced, and prognosis is worsened.

### Management

Mechanical ventilation was instituted in accordance with guidelines and was controlled so that each air exchange would be 6–8 mL/kg (ideal body weight), not to exceed 10 mL/kg. At the first day, positive end-expiratory pressure (PEEP), peak inspiratory pressure (PIP) and tidal volume were recorded.

As described, there is controversy on the use of corticosteroids in ARDS. All of the patients in our study received corticosteroid treatment. In the high-dose corticosteroid therapy group, intravenous (i.v.) methylprednisolone was infused at a dose of 1000 mg/day for the first 3 days and from the 4th day, a moderate dose (2 mg/kg/day) was administered and gradually tapered off over a period of 1.5 − 2.0 months. In contrast, the low-dose group was treated with a methylprednisolone dose of 0.5–1.0 mg/kg/day from the first day and the drug was tapered off over a period of 1.5–2.0 months. Multiple respiratory specialists made a comprehensive decision whether or not to institute high-dose corticosteroid therapy based on clinical findings at the time of admission. Specifically, high-dose corticosteroid therapy was used when they felt the disease might respond to corticosteroids.

### Statistical analysis

The primary outcome was the 60-day mortality rate. The secondary outcome was the number of ventilator-free days (VFD) [21], over a 28-day period, the total number of organ-failure-free days (OFFD) [21], and whether or not VAP occurred during that period.

Patients were divided into two groups depending on whether or not they had undergone high-dose corticosteroid therapy (high-dose corticosteroid vs. low-dose corticosteroid group). The chi-squared test (without Yates correction) or Fisher’s exact test was used to compare categorical variables. Differences in the means of continuous variables were tested by Student’s *t* test or Welch’s *t* test and confirmed using the Mann-Whitney *U* test. Propensity scores were estimated for the efficacy high-dose corticosteroid therapy. Compounding factors were age, sex, whether an infectious cause was involved, direct factors or indirect factors, APACHE II score, SOFA score, McCabe score, PaO2/FiO2 ratio, severity based on the Berlin definition, and blood test results at admission (white blood cell counts, CRP, LDH, albumin, platelet counts). Propensity scores were estimated using a logistic regression model. Receiver operator characteristic curves were plotted based on the calculated propensity score to determine precision. The propensity score was used with inverse-probability-of-treatment weighted (IPTW) methods and 60-day mortality and VFD were analyzed as dependent variables. In IPTW methods, patients are weighted by the inverse probability of receiving high-dose corticosteroids. Using these methods we could reduce or eliminate confounding by those measured covariates [22]. All analyses were performed using IBM SPSS Statistics ver22.

## Results

### Patient characteristics

**Fig. 1:**
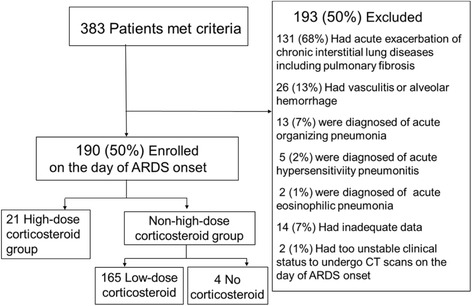
Study flowchart. *ARDS* acute respiratory distress syndrome, *CT* computed tomography

There were 21 patients who received high-dose corticosteroid therapy and 165 patients who received low-dose corticosteroid therapy (Fig. 1). The 68% cause of the ARDS were sepsis, pneumonia, or aspiration. LDH at admission was significantly higher in the high-dose corticosteroid therapy group, while CRP and PEEP was significantly higher in the low-dose group. There were no other differences between the two groups (Table 1). There were no significant differences in general severity, the extent of multiple organ failure or lung injury, or the extent of fibroproliferative changes on HRCT scans between the two groups as determined from the APACHE II score, SOFA score, and HRCT score at admission.

**Table 1:** Characteristics of patients in the study

Variables	High-dose group	Low-dose group	*P* value
	(n = 21)	(n = 165)	
Age, years	70 (64–80)	77 (69–83)	0.135
Sex, male, *n* (%)	11 (47.8)	105 (63.6)	0.316
Cause of ARDS pulmonary, *n* (%)	15 (65.2)	101 (61.2)	0.363
Cause of ARDS infection, *n* (%)	16 (69.6)	111 (67.3)	0.408
PaO_2_/FIO_2_ ratio	107.0 (60.1–140.4)	106.8 (77.2–153.6)	0.179
white blood cells (/mm^3^)	12500 (8250–15200)	9700 (5300–15150)	0.538
CRP (mg/dL)	8.90 (5.33–21.02)	16.1. (9.59–25.00)	0.031
LDH (IU/L)	478.0 (326.5–699.5)	324.0 (250.0–435.0)	0.011
Platelets (×10^4^/mm^3^)	17.0 (9.6–29.0)	18.4 (11.6–25.1)	0.664
Albumin (g/dL)	2.8 (2.6–3.2)	2.9 (2.4–3.2)	0.634
PEEP (cmH_2_O)	8.0 (7.0–10.0)	8.0 (8.0–12.0)	0.022
PIP (cmH_2_O)^a^	18.0 (10.0–25.0)	22.0 (18.0–25.0)	0.109
Tidal volume (mL)^b^	450.0 (357.5–468.8)	420.0 (350.0–490.0)	0.520
ARDS severity mild/moderate/severe	0/11/10	15/78/72	0.354
HRCT score	257.0 (209.1–290.8)	209.9 (183.4–283.0)	0.214
APACHE II score	19.0 (16.0–25.5)	22.0 (18.0–25.0)	0.240
SOFA score	6.0 (5.5–9.5)	7.0 (5.0–10.0)	0.494
McCabe classification category 1/2/3	19/1/1	145/10/10	0.942

Data are presented as median (IQR), unless otherwise stated. *ARDS* acute respiratory distress syndrome, *PaO*_*2*_ arterial oxygen tension, *FiO*_*2*_ fraction of inspired oxygen, *CRP* C-reactive protein, *LDH* lactate dehydrogenase, *ALB* serum albumin, *PEEP* positive end-expiratory pressure, *PIP* peak inspiratory pressure, *CT* computed tomography, *APACHE II* Acute Physiology and Chronic Health Evaluation II, *SOFA* Sequential Organ Failure Assessment, *HRCT* high-resolution computed tomography

^a^High-dose group (n = 18), low-dose group (n = 122)

^b^High-dose group (n = 14), low-dose group (n = 119)

### Patient outcomes

The 60-day mortality rate was significantly higher in the high-dose corticosteroid therapy group (*P* = 0.031). There were no significant differences between the two groups in the rate of onset of VAP. The number of VFD by day 28 was significantly smaller in the high-dose corticosteroid therapy group (*P* = 0.021), and there were no significant differences in OFFD at day 28 between the two groups (Table 2).

**Table 2:** Outcomes of patients with acute respiratory distress syndrome (n = 186)

Variables	High-dose group	Low-dose group	*P* value
	(n = 21)	(n = 165)	
60-Day mortality	66.6%	41.9%	0.031
Ventilator-free days by day 28	0.0 (0.0–7.5)	10.0 (0.0–19.0)	0.021
Organ-failure-free days by day 28	71.0 (28.5–94.5)	97.0 (48.0–110.0)	0.087
Ventilator-associated pneumonia	9 (42.9%)	62 (37.6%)	0.639

Data are presented as median (IQR), unless otherwise stated

**Fig. 2:**
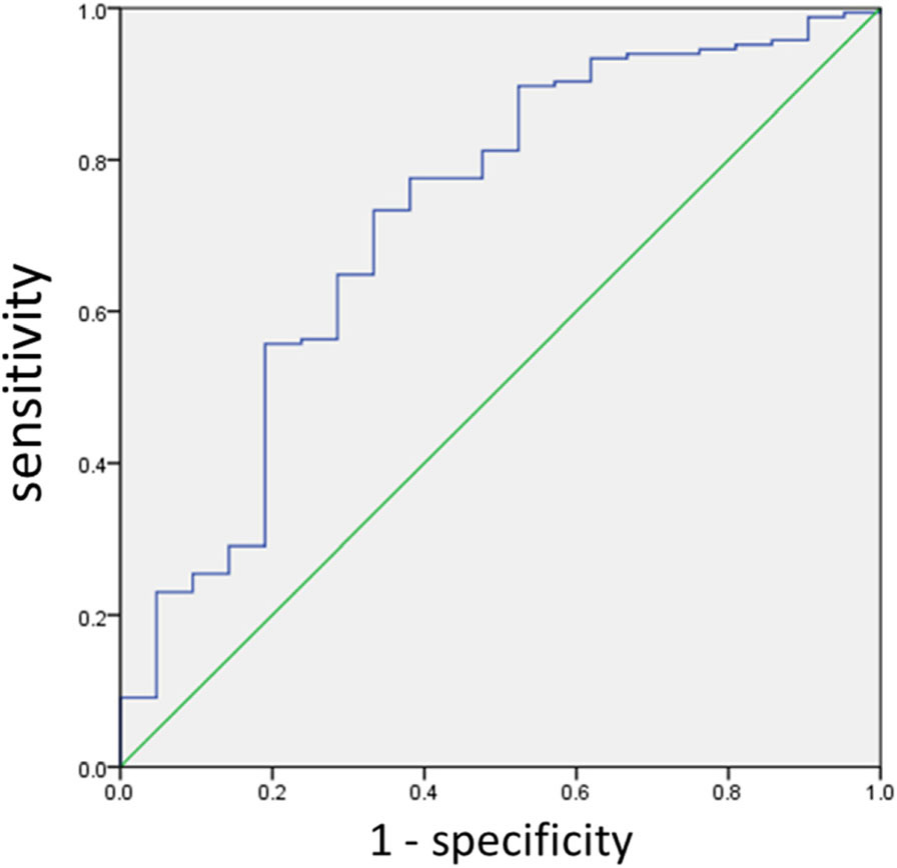
Receiver operating characteristic curve showing the prognostic value of the propensity score for high-dose corticosteroid therapy. The area under the curve is 0.723 (95% CI, 0.599–0.847)

We calculated the propensity scores on whether or not to conduct high-dose corticosteroid therapy based on the confounding factors at the time of admission. Propensity score precision was evaluated using a receiver operating characteristic curve; the area under the curve showed good precision equaling 0.723 (95% CI, 0.599–0.847) (Fig. 2). Inverse-probability score-based weighted methods were used to adjust multiple logistic regression analyses. When high-dose corticosteroid therapy was instituted, the number of VFD significantly decreased (OR, 95.63; 95% CI, 1.74–5271.07; *P* = 0.026), and the 60-day mortality rate tended to be higher (OR, 2.54; 95% CI, 0.92–7.02; *P* = 0.072) (Table 3).

**Table 3:** Logistic regression with inverse probability of treatment weighting of 60-day mortality and ventilator-free days by day 28 in patients with acute respiratory distress syndrome (n = 186)

Outcome	Odds ratio	95% Confidence interval	*P* value
60-Day mortality	2.54	0.92–7.02	0.072
Ventilator-free days by day 28	95.63	1.74–5271.07	0.026

## Discussion

The most important finding from this study was that initiating treatment with high-dose methylprednisolone therapy followed by a tapering regimen led to prolonged ventilation and tended to worsen prognoses in our patients who had ARDS that was caused by infectious processes, in spite of there being no significant differences in disease severity at the time of diagnosis. Although there were differences in the dose and duration of administration, these results do not contradict previously reported results on the use of high-dose corticosteroids in ARDS [23]. In reporting on the efficacy of short-course high-dose corticosteroids, Bernard et al. did not observe an effect on mortality rates [8]. Others have reported similar results, but all of these are from the 1980s [9], and our study is the first to evaluate corticosteroid use in patients diagnosed with ARDS according to the Berlin definition. In addition, high-dose therapy followed by corticosteroid tapering has been little studied before in this patient population [4]. Compared to the 1980s, intensive care treatment has progressed significantly, with better mechanical ventilation technology. It is worth noting that in spite of these improvements, initiating treatment with high-dose corticosteroids and subsequent tapering can be associated with greater mortality. In contrast, with the exception of four patients, all the other patients were treated with low-dose corticosteroids. Administration of low-dose corticosteroids with a tapering regimen in the subacute phase of ARDS was shown to shorten the duration of mechanical ventilation in a large randomized control trial [4]. Meta-analyses up to this point [24] have shown that low-dose corticosteroids do reduce mortality rates and there have been no results that suggest outcomes would be worsened with this treatment [6, 7]. Although it may be reflected on the better mortality of low-dose group than that of high-dose one in our study, the mortality rate in the low-dose group was 41.9%, which is similar to numbers reported in the past [25–27]. Further investigation is needed to examine whether or not low-dose corticosteroids should be used in ARDS.

Controversy regarding the efficacy of corticosteroids in ARDS still needs to be addressed. In addition, the anti-inflammatory effects of corticosteroids have been reported as effective in continuous control of the SIRS that complicates ARDS [28]. More recently, it has been suggested that we need to reevaluate the optimal timing of administering corticosteroids based on the pathological phase of diffuse alveolar damage (DAD), which is a characteristic histopathological finding of ARDS [29, 30]. It has been suggested that the effectiveness of corticosteroids may depend on the extent of inflammation seen in each pathological phase of DAD. In other words, in the near future we may need to reevaluate the use of corticosteroids during the acute exudative phase seen within the first week of onset when inflammation is particularly exaggerated. Furthermore, higher-dose corticosteroids might be beneficial in some cases of ARDS such as drug-associated ARDS, in which lung injury is attributed to immunostimulatory effects. In this study, all but four patients were started on high-dose or low-dose corticosteroids from the time of diagnosis within one week of ARDS onset, and a tapering regimen was selected for continuous control of SIRS. We calculated the propensity score and by analyzing data using IPTW methods, background factors and severity were strictly adjusted for. This allowed us to determine that high-dose corticosteroids with a tapering regimen significantly increased the duration of mechanical ventilation. One point to note is that in certain cases, such as in eosinophilic pneumonia, corticosteroids are known to be effective and are definitely exceptions to ARDS﻿ diagnosis.

High-dose corticosteroid treatment has been reported to worsen the prognosis in septic shock and serious sepsis [31]. Compared to the low-dose group, high SOFA scores were maintained on day 3 and beyond in the group that received high-dose corticosteroids therapy. ARDS was due to infection in approximately 68% of the patients in our study, and so initial high-dose corticosteroid treatment might have affected the immune system and aggravated the causative infectious disease process, making it more likely to cause multi-organ failure. Addressing the concept of bio-trauma due to mechanical ventilation [32–34], as the numbers of VFD were lower in patients in the high-dose corticosteroid group, the prolonged mechanical ventilation would increase the susceptibility of secondary multi-organ failure risk due to bio-trauma.

There are several limitations to our study. First, it was a single site and a retrospective study. There are several missing values. Second, there are no global standards for treatment duration, dose, or administration methods in corticosteroid therapy. As mentioned above, a prospective investigation is necessary in order to determine the optimal duration, dose, and administration method of corticosteroid treatment. The third point is that although multiple physicians were involved in deciding upon treatment policies, it was not always the same physicians involved in deciding upon the treatment for all patients. For this reason, there may have been differences in the appropriateness of the treatment policies. However, the same group of physicians was involved in checking the appropriateness of the diagnoses and in that respect there was consensus. The fourth point is that the high-dose groups are extremely ﻿fewe﻿r than the control group. Finally, the patients treated with high-dose corticosteroids had higher acuity, which is reflected in the decrease in the adjusted vs. the non-adjusted odds ratio *P* values, thus it is possible that there may be further residual confounding due to unmeasured variables not accounted for in the adjusted analysis, which could explain the greater mortality.

## Conclusion

A tapering regimen after initiation of high-dose corticosteroids is likely to increase ventilator dependency and might worsen the prognosis of patients with ARDS diagnosed according to the Berlin definition. Corticosteroids should not be instituted based merely on the assumption that they “might”be effective. In respiratory failure that satisfies the Berlin definition, a differential diagnosis should be performed to carefully select those diseases in which corticosteroids will be effective.

## Abbreviations

APACHE: Acute Physiology and Chronic Health Evaluation
ARDS: Acute respiratory distress syndrome
BAL: Bronchoalveolar lavage
cfu: Colony-forming units
CRP: C-reactive protein
CT: Computed tomography
DAD: Diffuse alveolar damage
FiO_2_: Fraction of inspired oxygen
HRCT: High-resolution computed tomography
IPTW: Inverse-probability-of-treatment weighted
LDH: Lactate dehydrogenase
OFFD: Organ failure-free days
PaO_2_: Arterial oxygen tension
PEEP: Positive end-expiratory pressure
PIP: Peak inspiratory pressure
SIRS: Systemic inflammatory response syndrome
SOFA: Sequential Organ Failure Assessment
VAP: Ventilator-associated pneumonia
VFD: Ventilator-free days
